# Tenofovir Pre-exposure Prophylaxis for Pregnant and Breastfeeding Women at Risk of HIV Infection: The Time is Now

**DOI:** 10.1371/journal.pmed.1002133

**Published:** 2016-09-27

**Authors:** Lynne M. Mofenson

**Affiliations:** Elizabeth Glaser Pediatric AIDS Foundation, Washington, D.C., United States of America

## Abstract

In this Perspective, Lynne Mofenson discusses the implications of Mugwanya and colleagues' findings for protection of women against HIV infection during breastfeeding.

Pre-exposure prophylaxis (PrEP) using either daily oral tenofovir disoproxil fumarate (TDF) or co-formulated TDF/emtricitabine (TDF/FTC) has been shown in clinical trials to be effective for prevention of HIV acquisition in men who have sex with men, heterosexual men and women, and persons who inject drugs [1]. However, unfortunately, the relevant clinical trials excluded pregnant or breastfeeding women; the trials included frequent pregnancy testing, and PrEP was discontinued if pregnancy was recognized (generally at gestation months 1–2).

In 2015, the World Health Organization (WHO) recommended PrEP as “an additional prevention choice for people at substantial risk of HIV infection, as part of combination HIV prevention approaches,” defining “substantial risk” as HIV incidence >3 per 100 person-years in the absence of PrEP [2]. Based on the available data, pregnant and lactating women residing in sub-Saharan Africa clearly meet this definition. In a systematic review and meta-analysis including data from 19 cohorts representing 22,803 total woman-years, the pooled HIV incidence during the pregnancy/postpartum periods was 3.8/100 woman-years (4.7 and 2.9/100 woman-years in the pregnancy and postpartum periods, respectively), with a 3.6% pooled cumulative incidence in African countries compared to 0.3% in non-African countries [3].

However, according to current WHO guidelines, “further research is needed to fully evaluate PrEP use during pregnancy and breastfeeding.” The approved drug label for TDF states that the drug “should be used during pregnancy only if clearly needed…Because of both the potential for HIV-1 transmission and the potential for serious adverse reactions in nursing infants, mothers should be instructed not to breastfeed if they are receiving [the drug]” [4]. Thus, there is a critical need to examine the safety of PrEP in HIV-uninfected pregnant and breastfeeding women (particularly adolescent and young women) and their infants.

The accompanying Research Article by Kenneth Mugwanya and colleagues in *PLOS Medicine* significantly contributes to the accumulating safety data for PrEP in breastfeeding women [5]. This documents a prospective study of daily TDF/FTC in 50 HIV-uninfected breastfeeding women between 1–24 weeks postpartum; the drug combination was provided to women for ten consecutive days and then discontinued. Tenofovir, the active drug moiety of TDF, has very low bioavailability, and it is therefore administered as the water-soluble prodrug to increase permeability across epithelial barriers and, once absorbed, is rapidly converted to tenofovir. Because it is the active drug that is present in maternal blood, it would be expected that there would be low penetration of tenofovir across the blood–breast epithelial barrier into breast milk. Consistent with this hypothesis, breast milk tenofovir concentrations were extremely low (3.2 ng/mL) in Mugwanya and colleagues’ study; FTC concentrations were somewhat higher (212.5 ng/mL), as has been seen for lamivudine, abacavir, and zidovudine [6–8]. In infant plasma, tenofovir was below the limit of detection in 46 (94%) of 49 samples; 47 of 49 (96%) infant samples had detectable FTC. Based on breast milk concentrations, breastfeeding infants would have exposures to TDF 12,500-fold lower, and to FTC 200-fold lower, than those achieved with pediatric therapeutic dosing (<0.01% and 0.5% of therapeutic dose, respectively). These data confirm and extend other studies that have reported very low concentrations of tenofovir detectable in breast milk, and strongly suggest that TDF and TDF/FTC can safely be given to breastfeeding women without putting their infants at risk of adverse effects [8–11].

The data provided by Mugwanya and colleagues’ study and others are important because sustained high HIV incidence among adolescent and young women in sub-Saharan Africa constitutes a significant public health emergency [12,13]. HIV prevalence is 1.7 times higher among young women in sub-Saharan Africa than in young men and 8 times higher among females than males aged 15–19 years in South Africa; among adolescents in sub-Saharan Africa, 71% of new HIV infections are among females [13]. Pregnancy rates in these young women are also high. Sub-Saharan Africa has the highest prevalence of pregnancy in women aged 15–19 years globally; births to teenage mothers account for more than half of all births, an estimated 101 births per 1,000 women aged 15–19 years [14]. Being a pregnant or lactating woman in sub-Saharan Africa is associated with a substantial risk of HIV acquisition, and acute HIV infection during pregnancy or lactation is associated with high rates of mother-to-child HIV transmission [3,15–17].

Although limited, most data on TDF during pregnancy are from HIV-infected women receiving combination antiretroviral therapy (ART), with the majority of studies in HIV-infected women on ART and their infants showing no adverse effects of TDF exposure [18–20]. However, the balance of benefits and risks of using TDF in HIV-infected pregnant women, in whom there is known risk of morbidity and mortality without therapy, differs from use in HIV-uninfected women, in whom the drug is being used for prophylaxis rather than treatment of infection. Additionally, because pregnancy outcomes among HIV-infected women, even those receiving ART, are worse than in HIV-uninfected women, comparability of data from the HIV-infected to the uninfected population has limitations and likely provides a worst-case scenario in terms of adverse events [21].

TDF has been used for prevention of perinatal hepatitis B virus (HBV) transmission in HBV-infected pregnant women with high concentrations of HBV DNA, for whom the risk of perinatal HBV transmission is high even when the infant receives hepatitis B immunoglobulin and HBV vaccine [22]. In these instances, TDF, given as a single drug, is initiated in the third trimester of pregnancy and usually (but not always) stopped 1–2 months postpartum. Studies in HBV mono-infected pregnant women demonstrated adverse event rates much lower than those seen in HIV-infected women, and no significant differences in pregnancy outcomes were observed between TDF and no drug exposure [23,24].

TDF and TDF/FTC have been evaluated in oral PrEP randomized, controlled clinical trials that included non-pregnant women of child-bearing age, in which pregnancies have occurred in women receiving PrEP at the time of conception. Two of these trials have reported on pregnancy outcomes in such women, reporting no difference in adverse pregnancy outcomes between active and placebo arms [25,26]. However, in these trials, PrEP was discontinued after pregnancy was recognized, and, in the VOICE trial, adherence to PrEP was suboptimal [27].

Given that young women, particularly if pregnant or breastfeeding, in sub-Saharan Africa experience some of the highest incidence rates of HIV infection globally, the benefits of HIV prevention in pregnant and breastfeeding women and their infants in these regions, given the currently available evidence, seem to clearly outweigh the risks observed to date. A recent decision analytic modelling study found that the HIV prevention benefit of providing PrEP to pregnant and breastfeeding women in sub-Saharan Africa outweighed even a substantially increased risk of preterm birth (as high as 30%) [28]. For HIV-serodiscordant couples in which the infected partner starts ART, additional prevention options such as PrEP are still needed, because there is residual HIV transmission risk during the first 6 months of ART until viral suppression is achieved [19,29].

Although WHO calls for further research, current WHO guidelines are permissive for use of PrEP during pregnancy and breastfeeding, noting growing evidence for safety from maternal HIV and HBV studies [2]; WHO is currently reviewing data on safety of PrEP in pregnancy and lactation and will provide more detailed guidance in the near future. However, although it will be important to collect additional safety data, the weight of the existing evidence does not support further delay in implementing TDF PrEP for pregnant and breastfeeding women at high risk of HIV acquisition. Those women on PrEP who become pregnant or are lactating should not have to stop an effective HIV prevention intervention.

## Abbreviations

ART: antiretroviral therapy
FTC: emtricitabine
HBV: hepatitis B virus
PrEP: pre-exposure prophylaxis
TDF: tenofovir disoproxil fumarate
